# GENES, LIPIDS, AND ALZHEIMER DISEASE RISK IN FAMILIES WITH EXCEPTIONAL LONGEVITY

**DOI:** 10.1093/geroni/igae098.0030

**Published:** 2024-12-31

**Authors:** Rong Cheng, Laura Xicota, Sandra Barral Rodriguez, Lawrence Honig, Gary Patti, Joseph Lee

**Affiliations:** Columbia University, New York, New York, United States; Columbia University Irving Medical Center, New York, New York, United States; Columbia University, New York, New York, United States; Columbia University Irving Medical Center, New York, New York, United States; Washington University in St. Louis, St. Louis, Missouri, United States; Columbia University, New York, New York, United States

## Abstract

Clinical studies have consistently shown that various classes of lipids are involved in the early stages of Alzheimer’s Disease (AD). To better understand the biological processes involved, we examined three layers of omic data from the Long Life Family Study (LLFS), which aimed to investigate the biological basis of familial exceptional longevity. Using 3,059 US participants, we performed a multi-stage analysis examining whole genome sequencing, transcriptomic, and lipidomic data. In addition, we examined two independent datasets for confirmation: the Alzheimer’s Disease Neuroimaging Initiative (ADNI) cohort, representing the general population; and the Multiomic Studies of Alzheimer’s Disease in Adults with Down Syndrome (omicsADDS), representing the high-risk Down Syndrome cohort. First, to identify biologically relevant lipids, we identified 18 lipids of 188 were shown to be highly heritable and were associated with AD risk. Our genome search of 18 lipids yielded two loci: 11q12.2 included MYRF, TMEM258, FEN1, FADS1, FADS2, and FADS3 as candidate genes; while 20p12.1 included SPTLC3 and LINC01723. After excluding genes not differentially expressed between affected and unaffected individuals, genes on 11q12.2 were associated with five lipids, including classes of triacylglycerol, phosphatidylcholine, and lysophophatidylcholine. In contrast, genes on 20p12.1 were associated with the class of ceramide non-hydroxy fatty acid-sphingosine. Lastly, we confirmed the 11q12.2 associations observed in LLFS in the ADNI and omicsADDS datasets. In sum, we showed that 11 AD-associated lipids were associated with genes on 11q12.2 in LLFS and were confirmed in the general population and the high-risk cohort.

